# Are octahedral clusters missing on the carbon energy landscape?†

**DOI:** 10.1039/c8na00013a

**Published:** 2018-09-11

**Authors:** Tomas Lazauskas, Alexey A. Sokol, Scott M. Woodley

**Affiliations:** University College London, Department of Chemistry Kathleen Lonsdale Building, Gower Street London WC1E 6BT UK t.lazauskas@ucl.ac.uk a.sokol@ucl.ac.uk scott.woodley@ucl.ac.uk

## Abstract

We report a new class of carbon nanostructures at a lower sub-nano end of the size scale with a surprising stability, as compared to the well-known carbon fullerenes. The octahedral carbon clusters contain tetragonal rings, which, in spite of a common belief, prove to be an energy efficient means of plying graphene sheets to make three-dimensional spheroid shapes, similar to fullerenes. The two families of structures are shown to be competitive at small sizes (∼20 atoms) at room temperature, and for higher temperatures, at both small and large sizes (>200 atoms). Our calculations demonstrate that both vibrational and electronic spectra of these cluster families are similar, which thus might cloud their experimental identification. However, there is a sufficiently strong shift in vibrational frequencies below 160 and in the range of 600–800 cm^−1^, which should help to identify different types of carbon clusters experimentally. We propose octahedral clusters and other structures containing tetragonal rings as viable structural elements and building units in inorganic chemistry and materials science of carbon along with fullerenes.

## Abstract 2

A computational discovery of a new structural form of carbon competitive with fullerenes at the small size end of the nanoscale.

## Graphical abstract

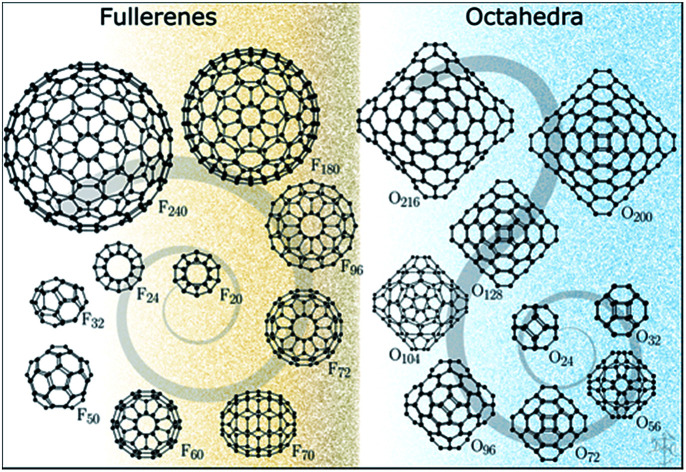

Many recent studies in the field of materials science and condensed matter physics have explored the possibility of using three-dimensional tailored building blocks made of carbon allotropes to create new carbon materials with tunable thermal and mechanical (in some cases stronger than diamond) properties.^1–8^ One of the synthetic routes to such materials is introducing nanosized fullerene-like spheroids into crystalline and amorphous structures,^9,10^ which can be thought of as superatoms, also known as secondary building units.^11,12^ This originates from a general belief in the carbon community that tetragonal, or four-membered rings in carbon structures are strongly energy penalised and five- and six-membered configurations are favoured instead, which has been strongly corroborated by the discovery of the Buckminster fullerene in 1985.^13^

The five-membered ring structural motif is in fact quite unique to carbon and similar covalently bonded compounds. It cannot, however, be adopted by polar binary compounds, which avoid bonding between likewise charged ions. The first fullerene-like structure in this class of materials was discovered by Behrman^14^ in 1994 using molecular dynamics simulations and empirical potentials for ZnO. The bubble-like structure is composed of twelve Zn and twelve O atoms, has a point symmetry of *T*_h_, and is typically referred to as a sodalite cage as its shape can be found in the cationic framework of sodalite. This predicted structure for the (ZnO)_12_ cluster proved to be just the first of a large family of perfect octahedral bubbles, in which each atom has a coordination of three, linking together six and four-membered rings. The most symmetrical structures in this family have four-membered rings occupying the truncated vertices of the octahedron whereas the octahedron's faces are composed of hexagonal tiles. These octahedral clusters are analogous to fullerenes, which have five-membered rings at the vertices of icosahedrons. Conceptually, both families of clusters can be created by cutting and folding hexagonal graphene sheets, so that the edges of these sheets form four- or five-membered rings that are necessary to make a perfect polyhedron with as little disruption to the hexagonal pattern as possible. These smaller 4- and 5-membered rings provide the curvature in the third dimension and represent the set of defects required for a finite hexagonal sheet with only 3-coordinated atoms. According to Euler's polyhedron formula, octahedral clusters will have six four-membered rings^15,16^ and fullerene clusters will have twelve five-membered rings.^17^

In this paper, we datamine Behrman's octahedral structures and, on the carbon energy landscape, show that their stability is very similar to that of fullerenes. Thus, we disprove the old belief that carbon does not form tetragonal rings and open a new class of plausible carbon structures amenable to future fundamental and applied studies.

In our work, we explore the resultant enriched carbon landscape that is defined by the state of the art density functional theory (DFT) and empirical potentials, all agreeing on the competitiveness of octahedral clusters with fullerenes.

The structures for this study (Fig. 1) have been datamined from two sources. The fullerene configurations (F) were obtained from the fullerene web-based library maintained by N. Frederick,^18^ which in turn hosts the fullerene geometries from the fullerene library of M. Yoshida. The octahedral structures (O) are based on those reported in ref. ^19–21^ (with appropriately scaled bond lengths). We note that for binary compounds, the so-called double bubble (DB) configurations are energetically competitive structural motifs;^22^ however, our initial calculations have shown that carbon DB configurations are much higher in energy than their single bubble counterparts and, therefore, were not included in this work. Nonetheless, we note that multilayered fullerenes were identified before^23^ and should be considered. Conversely, a one-layer bowl-like structure composed of 7 six-membered rings where atoms along the rim of the bowl have a reduced coordination of two, according to our DFT results, has a lower energy than both F_24_ and O_24_, thus indicating that for very small sizes there is significant competition between the formation of five-membered rings and reduction in coordination.

**Fig. 1:**
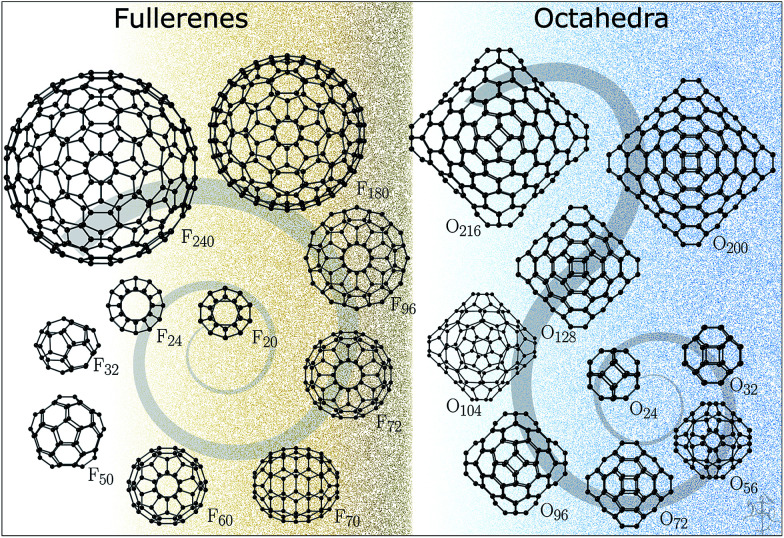
Fullerene and octahedron clusters studied in this work.

The energy of the investigated clusters was assessed at semiclassical and quantum mechanical density functional theory levels. All the structures were fully relaxed and the relative stability of each cluster was determined with respect to the optimised graphite structure using the corresponding level of theory.

To perform calculations at the semiclassical level, the General Utility Lattice Program (GULP)^24,25^ has been employed using two popular types of interatomic potentials (IP), namely Tersoff's bond-order potential, which has previously been successfully used to model small fullerene structures,^26^ and a general purpose Dreiding force field for carbon compounds.^27^ Throughout this paper we will refer to these two types of calculations as G1 and G2, respectively.

The quantum mechanical density functional theory (DFT) calculations were carried out using the well-known Fritz Haber Institute *ab initio* molecular simulations package (FHI-aims),^28^ which employs the local-density approximation (LDA), generalized gradient approximation (GGA) and hybrid functionals with predefined basis sets. We define A1 as the FHI-aims simulation using LDA and the light basis set, A2 – LDA with a tight basis set, A3 – GGA with a light basis set, A4 – GGA with a tight basis set, A5 – GGA with a tight basis set using the many-body dispersion (MBD) method,^29,30^ and, finally, A6 – hybrid functional PBE0,^31,32^ which includes 25% Hartree–Fock-like electron exchange, by performing single point energy calculations with a light basis set. When employing A1–A5, the structures are fully relaxed, whereas for A6 we use the atomic structure found using A4.

The computational settings for GULP, FHI-aims and the vibrational frequency calculations are provided in the ESI.†

As the first stage of our investigation, we have geometrically optimised the datamined structures at the semiclassical and DFT levels of theory and compared the structures in terms of their potential energy.

Calculations at both levels of theory showed the expected analogous behaviour for relative energies of fullerene (F) and octahedral (O) clusters with respect to the graphene sheet (Δ*E*): in most cases F clusters have a lower energy than O.

Unexpectedly, for smaller sizes at the DFT level of theory, octahedral clusters prove to be competitive with similarly sized fullerene clusters (Fig. 2(b)), *cf.* F_24_ and O_24_, which was not seen on the less accurate semiclassical potential energy surfaces (PES) (Fig. 2(a)).

**Fig. 2:**
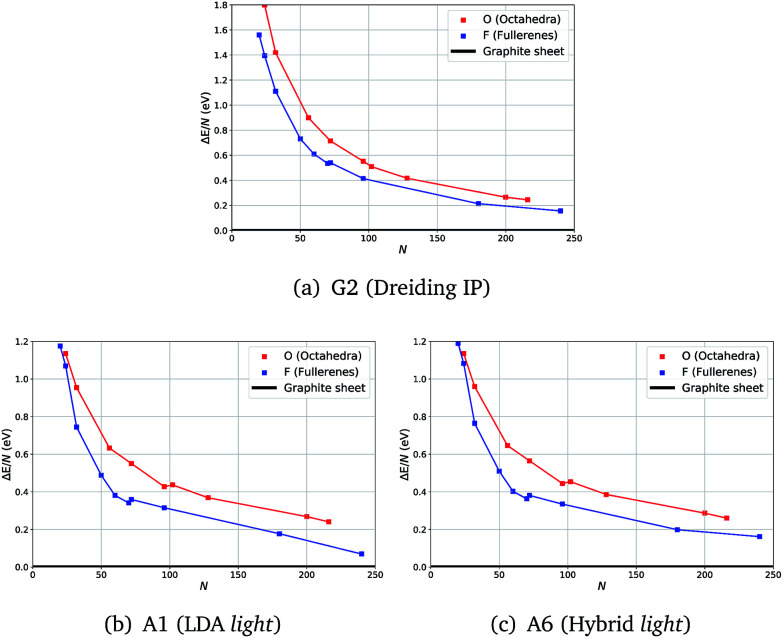
Energies with respect to the graphene sheet (Δ*E*) per atom of fullerene and octahedra clusters using three levels of theory.

From our semi-classical calculations, an approximately constant energy difference is observed between the curves for F and O, whereas from our DFT results they converge at smaller sizes. Moreover, although there are kinks in both DFT curves, between F_70_ and F_72_ and between O_96_ and O_104_, only one less pronounced kink appears between F_70_ and F_72_ for G2.

We note that the choice of a functional or larger basis sets did not show a significant effect on the relative stabilities of F and O clusters – the same trend is observed in all our tests.

For example, in Fig. 2 there are no noticeable differences in the profiles of the energy curves between those obtained using LDA (Fig. 2(b)) and those using a higher level of theory (Fig. 2(c)); whereas the curves are much smoother and run more in parallel for IP results (Fig. 2(a)). The computational time required significantly increases with the system size making the vibrational frequency calculations challenging already at the LDA level of theory. We, therefore, chose to compute the vibrational frequencies using the LDA level of theory along with the light basis settings (A1), whereas geometry optimization with a greater range of higher levels of theory is reported in the ESI.† All our results indicate a full consistency in the predicted relative stability of the carbon clusters studied and graphene.

Moreover, earlier calculations on carbon polymorphs have demonstrated unusual patterns in many-body dispersion contributions to their properties.^33^ Their influence on the relative stability of O and F clusters, however, proves to be only minor—for completeness we provide respective results together with a comparison of different levels of theory in the ESI.†

As the second stage of our investigation we turn our attention to the vibrational spectra of both the F and O clusters. The calculated frequencies may be used in the experimental characterisation of the systems and, in our work, we used them to obtain the Gibbs free energy. Vibrational frequencies for this study were computed *via* a script-based finite difference approach with FHI-aims employed to compute energies and first derivatives at the LDA level of theory.

In Fig. 3, for both types of systems, we show the average vibrational density of states (DOS), where each state has been Gaussian smeared. Remarkably, the DOS plots of the two classes of clusters are very similar: a gradual increase up to ∼700 cm^−1^, a minimum around 1000 cm^−1^ and a second maximum around 1380 cm^−1^.

**Fig. 3:**
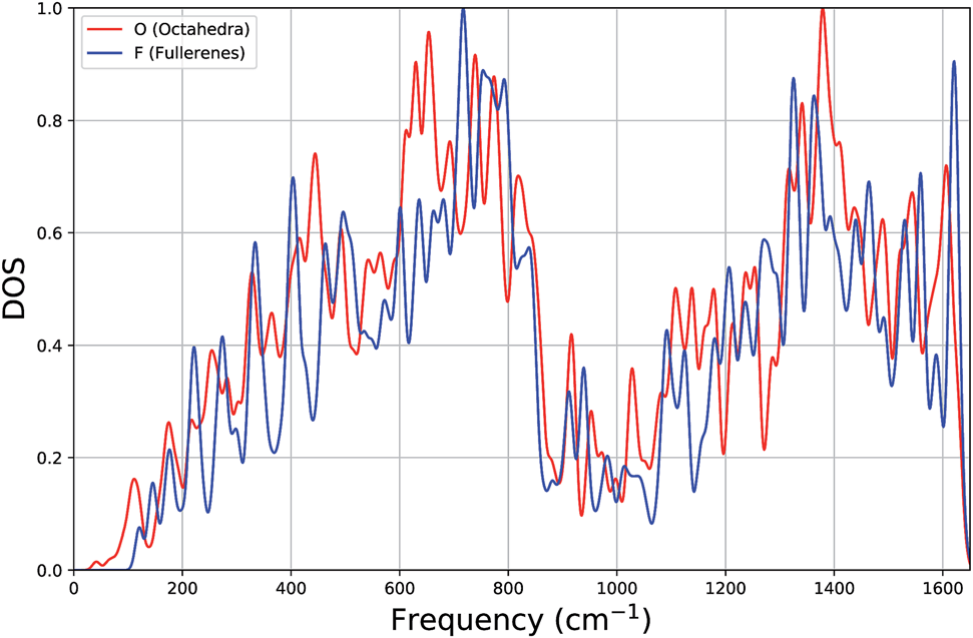
Average density of states (DOS) plot of vibrational frequencies of octahedron and fullerene clusters.

Looking more closely for differences within the lower part of the frequency spectrum, the average DOS for octahedral clusters, when compared to that for the fullerenes, begin at a lower frequency, has smaller contributions (local peaks) at approximately 220 cm^−1^ and 400 cm^−1^, has larger contributions at 440 cm^−1^, but more significantly there is a larger contribution to the DOS from a band of local peaks between 600 and 700 cm^−1^, whereas the fullerenes has a larger contribution between 700 and 800 cm^−1^. We believe that the shift in the contributions below 160 cm^−1^ and within 600 cm^−1^ and 800 cm^−1^ (octahedral DOS at lower frequencies) is significant enough to be useful for experimental discrimination between different carbon clusters with configuration type F and O.

In Fig. 4 we have drawn the electronic density of states for the following representative pairs: *F_24_ and O_24_, F_32_ and O_32_, F_72_ and O_72_, *F_96_ and O_96_, and F_240_ and O_216_, in order to check whether it is possible to distinguish these two classes of clusters by comparing their electronic structures. In contrast to vibrational spectra, the electronic structures of similarly sized fullerene and octahedral clusters do not show any specific trends in HOMO and LUMO values or DOS. We note that our DFT calculations for F_20_, F_24_ and F_96_ were performed in a spin-polarized triplet state, which proved to be more stable than the corresponding open shell singlets or closed-shell solutions.

**Fig. 4:**
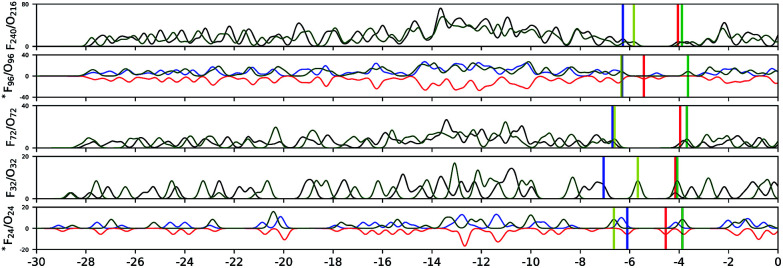
Electronic density of states (DOS) of F (fullerene) and O (octahedral) clusters. “*” marks the structures in the spin-polarized triplet state; black, blue and red lines represent the DOS of spin unresolved, spin up, and spin down projections, respectively; the blue and red vertical lines indicate HOMO (highest occupied molecular orbital) and LUMO (lowest unoccupied molecular orbital) energies for fullerene clusters; the green line shows the DOS, with the yellow and green vertical lines indicating HOMO and LUMO energies of octahedral clusters.

By calculating vibrational frequencies of the two classes of clusters, we were able to consider the relative cluster stabilities on the Gibbs free energy landscape. Thus, we are able to account for both zero point vibrational contributions and temperature dependent thermal effects. The vibrational contribution to the free energy is calculated using the standard expression,1
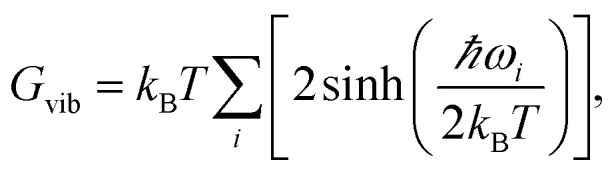
where *k*_B_ is the Boltzmann constant, *T* is the temperature, *ℏ* is the reduced Planck constant and *ω*_*i*_ represents the harmonic vibrational frequencies. We, however, omit translational and rotational contributions to the Gibbs free energy (*G*), as relatively large clusters would be expected to be immobilised in or on supports. Therefore, the Gibbs energy can be approximated as a sum of the potential energy (*U*) and the vibrational free energy (*G*_vib_) contributions:

2 *G* = *U* + *G*_vib_.

We have fitted an inverse power function to available free Gibbs energies for both types of clusters from 10 to 5000 K (that is in the range of the melting point of graphene). These functions *G*_O_ and *G*_F_, were used to estimate probabilities (eqn (3)) of finding the O clusters relative to F, which are plotted in Fig. 5.3
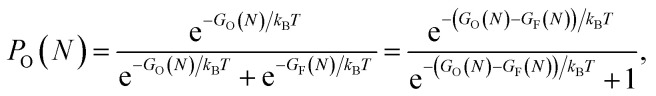
where *N* is the cluster size, *G*_O_(*N*) and *G*_F_(*N*) are the interpolated free energy functions between available data points for the O and F types of clusters, respectively, *k* is the Boltzmann constant and *T* is the temperature.

**Fig. 5:**
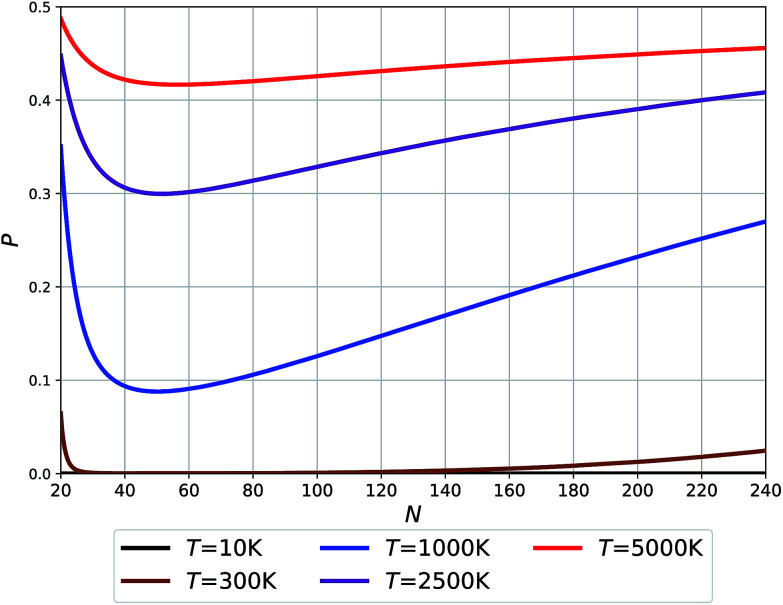
Probability of O clusters (relative to F) as a function of size at 10 K, 300 K, 1000 K, 2500 K, and 5000 K temperatures.

The temperatures in Fig. 5 relate to the synthesis or growth conditions under which such clusters can be obtained. At the lowest temperatures, we do not expect to find O clusters under thermodynamic equilibrium. (We note, however, that with a very low energy difference calculated for *N* = 24 between the two types of clusters, this prediction may change with the level of theory.) At 300 K only the smallest and largest O clusters will be seen with their fraction approaching ∼5%. Already at 1000 K, however, all O clusters across the full size range might be seen using an experimental technique capable of recognizing the difference between O and F cages. Finally, the high temperature synthesis should lead to an appreciable population of O clusters approaching 50% for the smallest sizes. The dip in the probabilities of finding O clusters at about *N* = 50–60 – *cf.* the Buckminster fullerene structure at *N* = 60 – is related to the maximum difference in their respective potential energies (see Fig. 2(b)) along with a moderate difference in their respective vibrational free energy contributions (see the integrated vibrational frequencies in the ESI†).

In this work we have presented evidence that octahedral clusters are energetically competitive with fullerene clusters, especially at the small and large cluster sizes. This contradicts the old belief that tetragonal, or four-membered rings in carbon structures are strongly energy penalised and, therefore, we should not restrict our models to those containing five- and six-membered configurations.

We have also shown a sufficient shift in vibrational frequencies, which might be useful for experimental discrimination between different types of carbon clusters, especially below 160 cm^−1^ and in the range of 600–800 cm^−1^.

## Conflicts of interest

There are no conflicts to declare.

## Supplementary Material

NA-001-C8NA00013A-s001
