# Supplementary material for: Are octahedral clusters missing on the carbon energy landscape?
Source: Nanoscale Adv. 2018 Sep 11;1(1):89–93. doi: 10.1039/c8na00013a (PMC9473225; doi:10.1039/c8na00013a)
Supplement: NA-001-C8NA00013A-s001 [file NA-001-C8NA00013A-s001.pdf]

Electronic Supplementary Material (ESI) for Nanoscale Advances.
This journal is © The Royal Society of Chemistry 2018

Are Octahedral Clusters Missing on the Carbon Energy Landscape?

Tomas Lazauskas,* Alexey A. Sokol,* and Scott M. Woodley*

University College London, Department of Chemistry, Kathleen Lonsdale Building, Gower Street, London WC1E 6BT, UK

E-mail: t.lazauskas@ucl.ac.uk; a.sokol@ucl.ac.uk; scott.woodley@ucl.ac.uk

Supporting Information Available

Computational Details

The main GULP parameters using Tersoff (G1) and Dreiding (G2) IP:

Table 1: Potentials and the main computational settings used within the GULP package.

Name	Potential	Convergence criterion based on			
		xtol (Å)	gtol	ftol ($eV/$Å)	switch rfo
G1	Tersoff	10^{-6}	10^{-4}	10^{-8}	10^{-2}
G2	Dreiding	10^{-6}	10^{-4}	10^{-8}	10^{-2}

The main FHI-aims parameters with respect to functionals used in the study:

Table 2: Functionals and the main computational settings used within the FHI-aims package. "-" notes that the default value or algorithm was used.

Name	Functional	Convergence criterion based on				
		Basis	charge density ($electrons$)	eigenvalues (eV)	total energy (eV)	forces ($eV/$Å)
A1	LDA	light	10^{-4}	10^{-3}	10^{-5}	10^{-3}
A2		tight	10^{-4}	10^{-3}	10^{-5}	10^{-3}
A3	GGA	light	10^{-2}	10^{-1}	10^{-2}	10^{-1}
A4		tight	10^{-4}	10^{-3}	10^{-5}	10^{-3}
A5	GGA+MDB	tight	-	-	-	-
A6	Hybrid	light	10^{-2}	10^{-1}	10^{-2}	10^{-1}

Vibrational frequencies have been calculated with FHI-aims using the LDA functional and *light* basis set. We have used the script-based finite difference approach from the FHI-aims package with the step size of δ=0.0025 Å.

Geometry Optimization

We report the summary of geometry optimisation calculations in Table 4 with respect to the graphite energy calculated at the same level of theory as it is given in Table 3. The lowest energy structure of 24 atoms (L_{24}) at the DFT level of theory is given in Figure 1.

Table 3: Total energy per atom of graphite at different levels of theory and computational settings, as described in Table 2.

Theory	Energy (eV)	Theory	Energy (eV)
G1	0.4324	A3	-1036.9925
G2	-7.3395	A4	-1037.0579
A1	-1029.0728	A5	-1037.2029
A2	-1029.1394	A6	-1037.0007

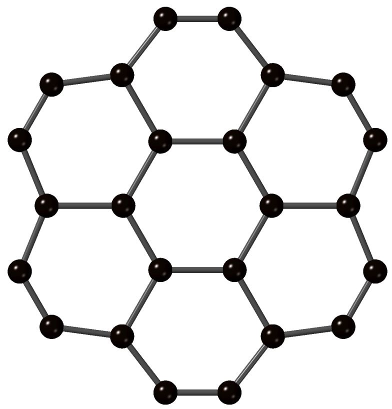

Figure 1: The lowest energy structure of 24 atoms - L_{24} - a one-layer convex structure containing 7 six-membered rings (terminated by two-coordinated atoms).

Density of States

The averaged vibrational density of states (DOS) have been calculated using the standard DOS expression with a Gaussian smearing function:

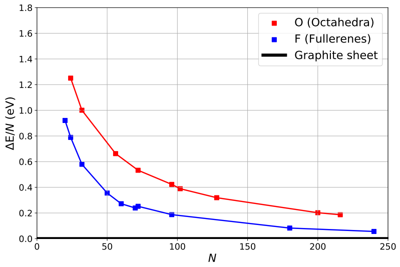

(a) G1 (Tersoff IP)

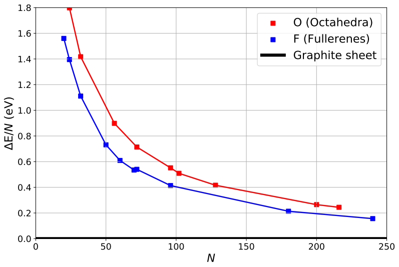

(b) G2 (Dreiding IP)

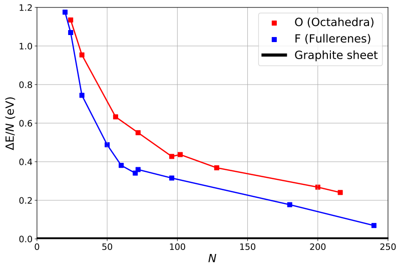

(c) A1 (LDA *light*)

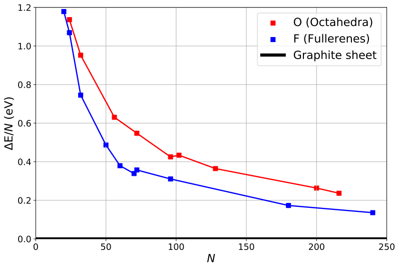

(d) A2 (LDA *tight*)

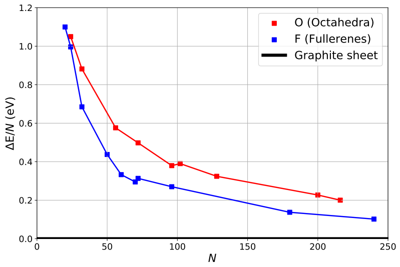

(e) A3 (GGA *light*)

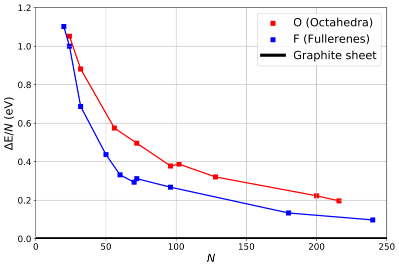

(f) A4 (GGA *tight*)

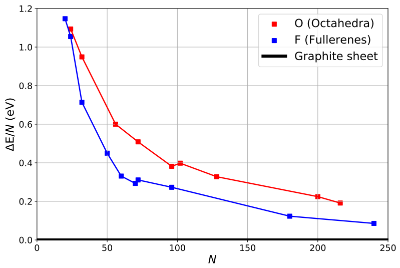

(g) A5 (GGA+MDB *tight*)

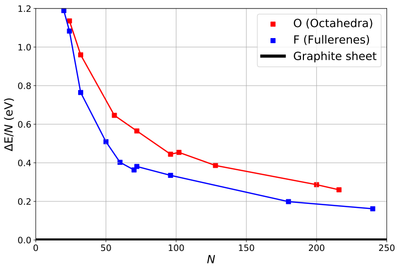

(h) A6 (Hybrid *light*)

Figure 2: Energy differences (Table 4) per atom between graphene sheet and the cluster structures with respect to the cluster size N. Yellow line represent layer structure, green - double bubbles, red - single bubbles and blue lines represent fullerene structures.

Table 4: Summary of energy differences with respect to the graphene sheet of optimised clusters' using different functionals and settings (as given in Tables 1 and 2). The subscript in name for layer, single bubble and fullerene configurations indicates the total number of atoms N, where for the double bubble configurations, this number indicates the number of atoms of the outer and the superscript - for the inner bubble atom counts respectively; "*" marks structures in spin-polarized triplet state. S is the cluster's symmetry point group, $\Delta E/N$ is the energy difference with respect to the graphite energy calculated at the same level of theory and using the same settings (Table 3) per atom, H-L is the HOMO-LUMO gap.

Name	S	G1	G2	A1		A2	A3	A4	A5	A6
		$\Delta E/N$ (eV)	$\Delta E/N$ (eV)	$\Delta E/N$ (eV)	H-L (eV)	$\Delta E/N$ (eV)	$\Delta E/N$ (eV)	$\Delta E/N$ (eV)	$\Delta E/N$ (eV)	$\Delta E/N$ (eV)
Layer										
L_{24}	D6h	-0.2064	1.1154	1.1451	2.205	1.2354	1.0845	0.9930		1.0635
Octahedral										
O_{24}	Oh	1.2506	1.7986	1.1349	1.232	1.2366	1.0498	1.0518	1.1356	1.0937
O_{52}	Oh	1.0006	1.4175	0.9538	0.729	1.0522	0.8820	0.8814	0.9597	0.9491
O_{56}	O	0.6624	0.8984	0.6327	1.078	0.7306	0.5761	0.5748	0.6459	0.6003
O_{72}	Oh	0.5328	0.7141	0.5506	1.569	0.6478	0.4981	0.4961	0.5651	0.5086
O_{96}	Oh	0.4227	0.5519	0.4274	1.475	0.5252	0.3796	0.3778	0.4446	0.3818
O_{104}	O	0.3891	0.5092	0.4371	0.294	0.5339	0.3901	0.3876	0.4539	0.3980
O_{128}	Oh	0.3191	0.4164	0.3686	0.889	0.4645	0.3243	0.3209	0.3861	0.3279
O_{200}	Oh	0.2021	0.2652	0.2682	0.142	0.3635	0.2271	0.2230	0.2866	0.2246
O_{216}	Oh	0.1864	0.2444	0.2405	0.984	0.3367	0.2003	0.1967	0.2601	0.1915
Fullerenes										
$^{*}F_{20}$	D3d	0.9209	1.5600	1.1732	0.423	1.2785	1.0998	1.1023	1.1891	1.1471
$^{*}F_{24}$	D6d	0.7889	1.3951	1.0657	0.228	1.1690	0.9963	1.0000	1.0830	1.0544
F_{32}	D3	0.5795	1.1108	0.7443	1.448	0.8454	0.6851	0.6868	0.7645	0.7139
F_{50}	D5h	0.3554	0.7311	0.4879	0.441	0.5868	0.4375	0.4371	0.5094	0.4498
F_{60}	Ih	0.2721	0.6093	0.3810	1.657	0.4794	0.3328	0.3319	0.4026	0.3312
F_{70}	D5h	0.2390	0.5348	0.3410	1.734	0.4388	0.2950	0.2935	0.3629	0.2933
F_{72}	D6d	0.2521	0.5402	0.3594	1.442	0.4573	0.3135	0.3119	0.3809	0.3111
$^{*}F_{96}$	D6d	0.1872	0.4136	0.3138	0.072	0.4107	0.2701	0.2677	0.3349	0.2731
F_{180}	Ih	0.0826	0.2137	0.1773	1.466	0.2732	0.1374	0.1338	0.1988	0.1232
F_{240}	Ih	0.0562	0.1561	0.1406	1.241	0.2359	0.1019	0.0977	0.1617	0.0858

$$\sum_k \left[\frac{1}{\sigma\sqrt{2\pi}} \sum_{i=1}^{N} \exp\left(\frac{\omega_i - \Omega_k}{2\sigma^2}\right)\right] /K, \tag{1}$$

where $\sigma = 10.0$ (Gaussian smearing parameter), ω_i is the vibrational frequency interval, Ω_k - the set of vibrational frequencies corresponding to a particular cluster k, and K - is the number of clusters of the same type, i.e. fullerenes or octahedra.

Fitting functions of the Gibbs free energy

The inverse power function (Equation 2) and its coefficients used for fitting free Gibbs energies for the O and F types of clusters at 10, 300, 1000, 2500 and 5000 K temperatures.

$$F(N) = \frac{a}{N^b} + c. \tag{2}$$

Table 5: The coefficients for the inverse power function to fit free Gibbs energies for the O and F types of clusters at 10, 300, 1000, 2500 and 5000 K temperatures.

T	Octahedral			Fullerene		
	a	b	c	a	b	c
10 K	8.36	0.61	-1036.95	27.69	1.06	-1036.83
300 K	8.18	0.60	-1036.96	27.90	1.06	-1036.83
1000 K	8.19	0.59	-1037.08	26.27	1.03	-1036.94
2500 K	8.61	0.59	-1037.65	23.05	0.97	-1037.51
5000 K	10.61	0.63	-1039.04	21.11	0.89	-1038.95

The constant c should correspond to the limit of the graphene sheet, cf. analyses in reference 17.

Integrated vibrational frequencies

We have individually integrated the vibrational frequencies for the O and F types of clusters in three ranges: 0-850 cm^{-1}, 850-1075 cm^{-1}, and 1075-1700 cm^{-1}.

Table 6: .

Cluster	0-850 cm^{-1}	850-1075 cm^{-1}	1075-1700 cm^{-1}
Octahedral			
O_{24}	36.87	4.11	25.00
O_{52}	47.00	10.10	32.89
O_{56}	83.00	14.16	64.81
O_{72}	109.00	14.96	85.92
O_{96}	145.75	18.21	117.68
O_{104}	160.90	21.60	122.97
O_{128}	194.26	28.37	155.23
O_{200}	310.62	40.92	242.11
O_{216}	332.48	41.35	267.56
Fullerenes			
F_{20}	24.70	5.07	24.16
F_{24}	32.00	4.46	29.49
F_{32}	46.00	5.00	39.00
F_{50}	74.91	9.61	59.33
F_{60}	91.99	8.00	74.00
F_{70}	105.98	9.84	88.07
F_{72}	108.86	11.92	89.00
F_{96}	146.99	15.88	118.97
F_{180}	276.41	35.40	221.98
F_{240}	367.86	49.76	295.89
